# Plant Tissue Culture and Secondary Metabolites Production

**DOI:** 10.3390/plants11233312

**Published:** 2022-11-30

**Authors:** Kalina Danova, Laura Pistelli

**Affiliations:** 1 Institute of Organic Chemistry with Centre of Phytochemistry, Bulgarian Academy of Sciences, Acad. Georgi Bonchev Str., bl.9, 1113 Sofia, Bulgaria; 2 Department of Agriculture, Food and Environment, University of Pisa, Via del Borghetto 80, 56124 Pisa, Italy

Plants have developed a complex biochemical system for interacting and coping with dynamic environmental challenges throughout their whole life. Plant secondary metabolites are specifically produced and accumulated in low quantities in response to numerous factors such as bacteria, viruses, fungi, nematodes, insects and herbivores, as well as to climatic factors and seasonal fluctuations, soil and water parameters, etc.

To optimize secondary metabolites production, plants have fine-tuned early signal systems for differentiating general mechanical damage from an attack by an insect/herbivore, for example. In addition, plants distinguish the degree of damage caused by herbivore feeding guilds, insect oral secretions, oviposition fluids, etc. The consecutive steps of the production of the respective defense secondary metabolites is mediated by cellular messengers and events such as metabolic changes, gene activation, jasmonic acid (JA) accumulation, kinase cascades, hydrogen peroxide production, cytosolic calcium ion fluxes, as well as membrane potential changes [1] and references cited therein. 

The key role of secondary metabolites for plant survival also underline the pharmacological roles that these substances play in mammalian organisms and hence their applicability in veterinarian and humanitarian medicinal practices. 

Plant cell tissue and organ cultures are based on the “totipotency” of the plant cell and its capability to regenerate up to a whole integral organism. The technique allows for the cultivation of separate cells, tissues, differentiated organs, or integral plants in a growth medium in sterile conditions and out of the indigenous natural environment of the plants. The contemporary development of the method has nowadays led to its routine use as a supplementary to conventional plant breeding for an array of applications such as the rapid and disease-free micropropagation of plantlets, independent of seasonal, climatic, and geographic factors; the rapid testing and practical introduction of new cultivars using conventional or genetic engineering selection approaches. An important and rapidly developing field of the application of plant biotechnology is its use for the yield of plant secondary metabolites, allowing for the standardization of the levels of the secondary metabolites produced due to the capability of tissue culture techniques to optimize culture conditions and obtain the desired environment for the production of the target compounds.

Considering the importance of plant secondary metabolites and the high relevance of the establishment of scientifically based approaches for their biotechnological production, we are pleased to present this Special Issue of *Plants* dedicated to “Plant Tissue Culture and Secondary Metabolites Production”. The present collection aims to provide readers with up-to-date research dedicated to the scientific accomplishments in the production of plant secondary metabolites of different chemical types through the development of plant cells, tissues, and organs in diverse in vitro culture systems.

We have received seven scientific research papers on tissue culture development and secondary metabolite production of medicinal and aromatic plants of different regions of the world.

Ramabulana et al. [2] established that the exogenous application of auxin (2,4-dichlorophenoxyacetic acid—2,4-D) and cytokinin (benzylaminopurine, BAP) could induce the manipulation of the metabolome of the callus culture of *Bidens pilosa* L. (Asteraceae), dominated by chlorogenic acids consisting of caffeoyl and feruloyl derivatives of quinic acid. Xanthones production in differentiated shoot cultures of the endangered *Gentianella lutescens* (Gentianeceae) was evaluated for the first time by Krstić-Milošević et al. [3] in an experiment focusing on the modification of the concentration of sucrose, sorbitol, and abiotic elicitors salicylic acid (SA), jasmonic acid (JA), and methyl jasmonate (MeJA). Wojtania and Mieszczakowska-Frąc [4] proposed an efficient biotechnological method to produce anthocyanins-rich planting material for selected genotypes of the Polish Culinary rhubarb ‘Malinowy’ cultivar. In a carrot (*Daucus carota*, Apiaceae) callus culture experimental design of a combination of the components of the Gamborg [5] and Murashige and Skoog [6] culture media, Oleszkiewicz et al. [7] established that the N concentration and the NO_3_:NH_4_ ratio affected carotenoid accumulation. In this work, changes to the medium other than N, such as microelements, vitamins, growth regulators, and sucrose, had no effect on callus growth and carotenoid accumulation. Mamdouh et al. [8] developed an in vitro protocol for micropropagation of *Lycium schweinfurthii* (Solanaceae). Investigations on genetic stability, phenolic, flavonoid, ferulic acid contents, and antioxidant activity were performed, leading to the selection of an effective protocol for the in vitro propagation of plant material with desired quality. Erst et al. [9] studied the combined effects of NO_3_, as well as NH_4_:K^+^ ratio and the cytokinins BAP and naphthylacetic acid (NAA) on the growth and production of total phenolics callus culture of *Rhodiola rosea* (Crassulaceae). The study of Pieracci et al. [10] on shoot cultures of halophyte *Artemisia caerulescens* L. (Asteraceae) demonstrated the potential of the tissue culture technique for both the ex situ conservation and production of essential oils and phenolic compounds of this species.

## Data Availability

Not appliable.
